# Unfavourable gender effect of high body mass index on brain metabolism and connectivity

**DOI:** 10.1038/s41598-018-30883-y

**Published:** 2018-08-22

**Authors:** Maura Malpetti, Arianna Sala, Emilia Giovanna Vanoli, Luigi Gianolli, Livio Luzi, Daniela Perani

**Affiliations:** 1 Division of Neuroscience, San Raffaele Scientific Institute, Milan, Italy; 2 Vita-Salute San Raffaele University, Milan, Italy; 3 Nuclear Medicine Unit, San Raffaele Hospital, Milan, Italy; 4 Metabolism Research Center and Endocrinology Unit, IRCCS Policlinico San Donato Milanese, Milan, Italy

## Abstract

The influence of Body Mass Index (BMI) on neurodegeneration in dementia has yet to be elucidated. We aimed at exploring the effects of BMI levels on cerebral resting-state metabolism and brain connectivity, as crucial measures of synaptic function and activity, in a large group of patients with Alzheimer’s Dementia (AD) (n = 206), considering gender. We tested the correlation between BMI levels and brain metabolism, as assessed by ^18^F-FDG-PET, and the modulation of the resting-state functional networks by BMI. At comparable dementia severity, females with high BMI can withstand a lower degree of brain metabolism dysfunction, as shown by a significant BMI-brain metabolism correlation in the temporal-parietal regions, which are typically vulnerable to AD pathology (R = 0.269, p = 0.009). Of note, high BMI was also associated with reduced connectivity in frontal and limbic brain networks, again only in AD females (p < 0.05 FDR-corrected, k = 100 voxels). This suggests a major vulnerability of neural systems known to be selectively involved in brain compensatory mechanisms in AD females. These findings indicate a strong gender effect of high BMI and obesity in AD, namely reducing the available reserve mechanisms in female patients. This brings to considerations for medical practice and health policy.

## Introduction

The interaction between Body Mass Index (BMI) and cognition in healthy status, and the role of BMI as a risk factor for dementia are complex and highly debated issues^1–3^. There is growing evidence that obesity (BMI >30 kg/m^2^) is linked to reduced cognitive functions in adults, particularly in executive, attention, and memory domains^3–5^. Epidemiological evidence on the risk of developing dementia in obesity is controversial. The complex interplay between BMI and the risk of developing cognitive decline might be modulated by age. Some studies reported a significant association between high BMI and cognitive deficits in young subjects^6^, and also a higher risk of developing dementia in midlife^7,8^. Despite the results in young adults and middle-aged subjects consistently suggest a negative effect of high BMI on cognitive functions, the evidence in late life is less clear. Epidemiological findings in elderly suggest a protective effect of high BMI against dementia (see for reviews^1–3^). This evidence merges in the “obesity paradox” hypothesis^9^, which again suggests a positive correlation between BMI levels and dementia risk in mid-life (age up to 60–65 years), but a protective effect of high BMI in late life (age 65–70 years or older) instead. To explain this discrepancy in late life, it has been suggested that low BMI may be a consequence of the loss of weight preceding the onset of dementia in older people^10,11^. An accelerated reduction in BMI during late life has been also reported to precede the clinical detection of dementia, while a slower decline in BMI has been associated with a reduced dementia risk^1^. Therefore, it is possible that low or declining BMI is a factor associated with the development of dementia rather than a risk factor. On the other hand, a lifelong high BMI has been equally suggested as a risk factor for cognitive decline and dementia by several studies, which reported an increased risk of developing cognitive decline in overweight or obese individuals across the lifespans^3,6,12^.

In spite of the extensive epidemiological data, neuroimaging studies are scarce and the neural mechanisms underlying the effect of BMI in dementia remain currently unknown. ^18^F-fluorodeoxyglucose Positron Emission Tomography (^18^F-FDG-PET) is a well-recognized tool to study brain dysfunction in dementia^13,14^. ^18^F-FDG-PET measures of resting-state brain metabolism, which is a direct *in vivo* index of synaptic function^13^, were applied in studies on modifiable risk and protective factors for dementia, although not on BMI. For example, there is large evidence for low education and occupation levels, low physical and leisure activities as risk factors for all dementia conditions^15,16^. On the other hand, life experiences such as high education^17–19^, occupation^17,18^ and bilingualism^20^ are known to be involved in the modulation of cognitive reserve inducing brain plasticity and structural changes in both healthy subjects and patients with neurodegenerative conditions^16^. These proxies of cognitive reserve seem to be crucial for processes related to the brain maintenance, leading to protection of brain integrity in aging and disease, and resulting in a higher brain reserve^16^. Several neuroimaging studies^17–20^ focused on brain reserve^16^, which is now recognized as one of the most powerful protective mechanism for cognitive decline and dementia^15^. These studies have suggested that high levels of reserve allowing cope with brain neurodegeneration, delaying the onset of clinical manifestation and leading to a higher tolerance to underlying pathology^17–20^.

We hypothesized possible modulatory effects of BMI on brain neurodegeneration and functional connectivity, and specifically a detrimental effect of high BMI and obesity on brain reserve, leading to major vulnerability to neuropathology. We thus studied a large group of patients with probable Alzheimer’s Dementia (AD), who underwent ^18^F-FDG-PET, in order to assess the effects of BMI levels on brain metabolism and connectivity, crucial measures of synaptic function and activity, while also considering the role of gender.

## Results

### Group analyses

**Figure 1:**
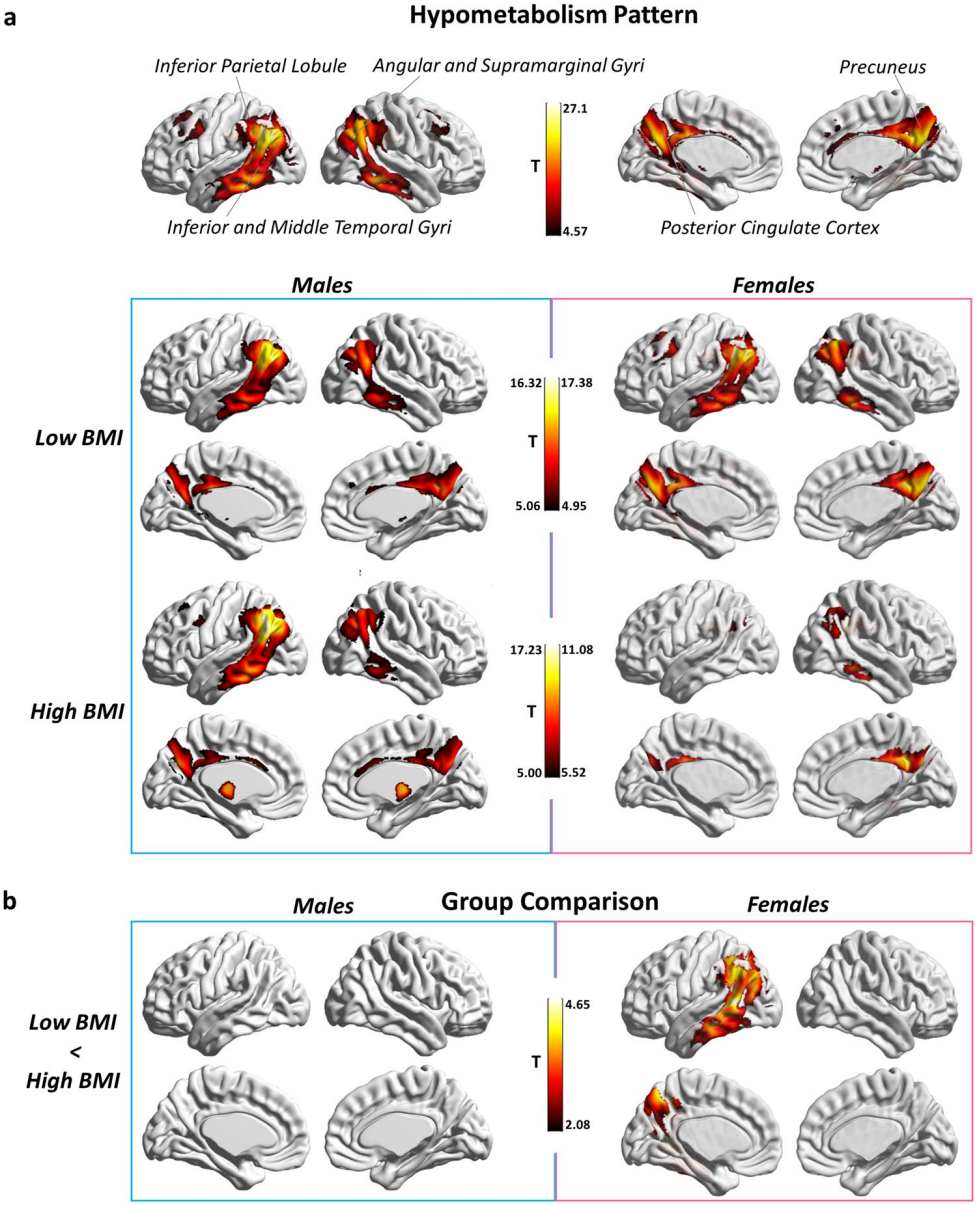
Brain hypometabolism AD patterns. (**a**) SPM-group analysis showing the typical AD brain hypometabolism pattern in the whole AD group. The analysis result is showed at p < 0.05 FWE corrected for multiple comparisons. (**b**) SPM-group analysis showing the typical AD brain hypometabolism pattern across low BMI vs. high BMI subgroups, separately for males and females (p < 0.05 FWE corrected for multiple comparison, k:100 voxels). (**c**) Results of the two-sample t-test comparisons between low BMI vs. high BMI gender subgroups. Significantly lower hypometabolism is present in precuneus/posterior cingulate cortex and parieto-temporal regions in females with high BMI as compared to females with low BMI (voxel-level p < 0.05 FDR corrected for multiple comparison, k = 100 voxels; cluster-level p < 0.05 FWE-corrected). No significant difference is revealed when comparing males with low vs. high BMI. The left side of the brain is shown on the left in the figure. Results are displayed on a 3D brain template using Brainnet toolbox^48^.

We first estimated AD-related hypometabolism by comparing AD patients with a large group of healthy controls. In the patients, the comparison showed the typical AD-like pattern (p < 0.05 FWE corrected for multiple comparison, k:100 voxels), with an extended and significant hypometabolism in the parietal-temporal cortex, and in the posterior cingulate cortex and precuneus (Fig. 1a). We then tested for differences in brain hypometabolism in AD patients with low BMI vs. high BMI, separately for female and male subgroups (p < 0.05 FWE corrected for multiple comparison, cluster extent (k) threshold at 100 voxels). The results showed a less severe and extended pattern of hypometabolism in the high BMI compared to the low BMI female subgroup and no differences between the male BMI subgroups (voxel-level p < 0.05 FDR corrected for multiple comparison, k = 100; cluster-level p < 0.05 FWE-corrected) (Fig. 1b,c). A multiple regression model confirmed the BMI x Gender interaction effect on brain metabolism in AD-signature brain regions (F = 6.185, p = 0.014; N = 206). There were no significant interaction effects between BMI and either age, education or APOE status on brain metabolism, neither in the whole AD group nor in gender subgroups.

### Metabolic correlation analysis

**Figure 2:**
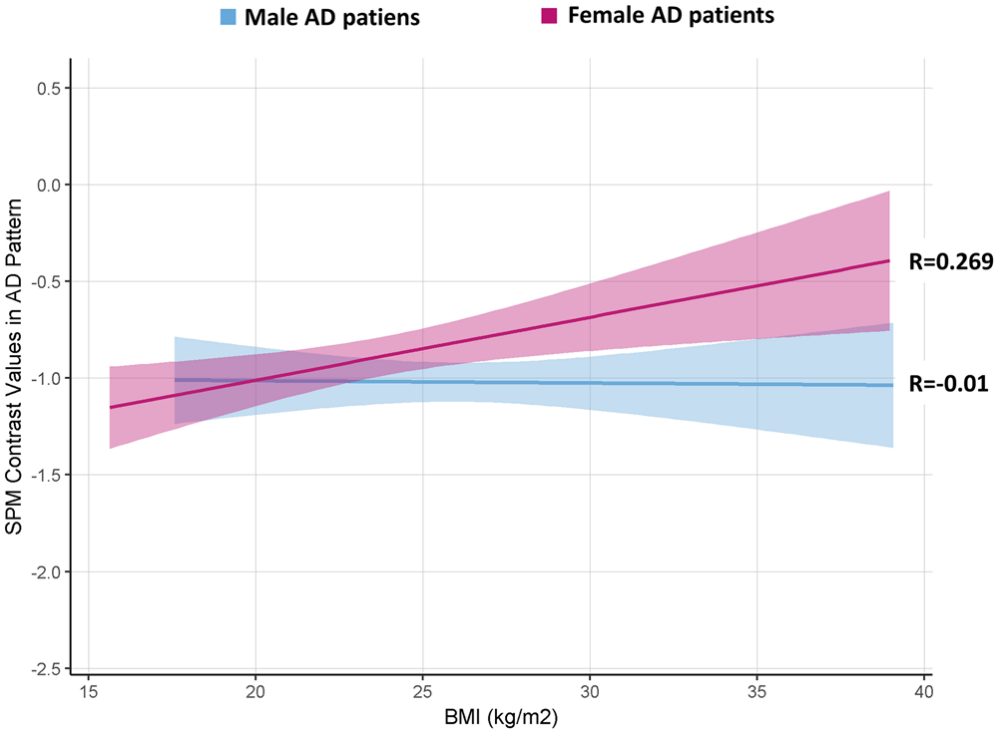
Correlation between BMI and metabolism in key AD regions. The graph displays the correlations between BMI (x axis) and SPM contrast values of ^18^F-FDG-PET glucose metabolism (y axis) in regions typically affected by AD, separately for females and males AD patients. A significant positive correlation is found for female AD patients (pink line; R = 0.269, p = 0.009; partial R = 0.224, p = 0.034); no correlation is reported for male AD patients (blue line; R = −0.010, p = 0.92; partial R = −0.055, p = 0.58; N = 112). Pink and blue shaded areas represent confidence intervals for regression lines’ slopes, in females and males AD patients, respectively.

The correlation analysis between BMI levels and brain metabolism, in regions typically affected by AD and representing the metabolic hallmarks of the disease, revealed no correlations in the whole AD group (R = 0.053, p = 0.45; partial R = 0.045, p = 0.52; N = 206). Considering gender, there was again no significant correlation in males (R = −0.010, p = 0.92; partial R = −0.055, p = 0.58; N = 112), whereas in females we found a significant positive correlation between BMI levels and glucose metabolism in the AD-typical affected regions (R = 0.269, p = 0.009; N = 94), also after controlling for age, disease duration, years of education and cognitive impairment, as measured by Mini-Mental State Examination (MMSE)(partial R = 0.224, p = 0.034; N = 94) (Fig. 2).

### Metabolic connectivity analysis

**Figure 3:**
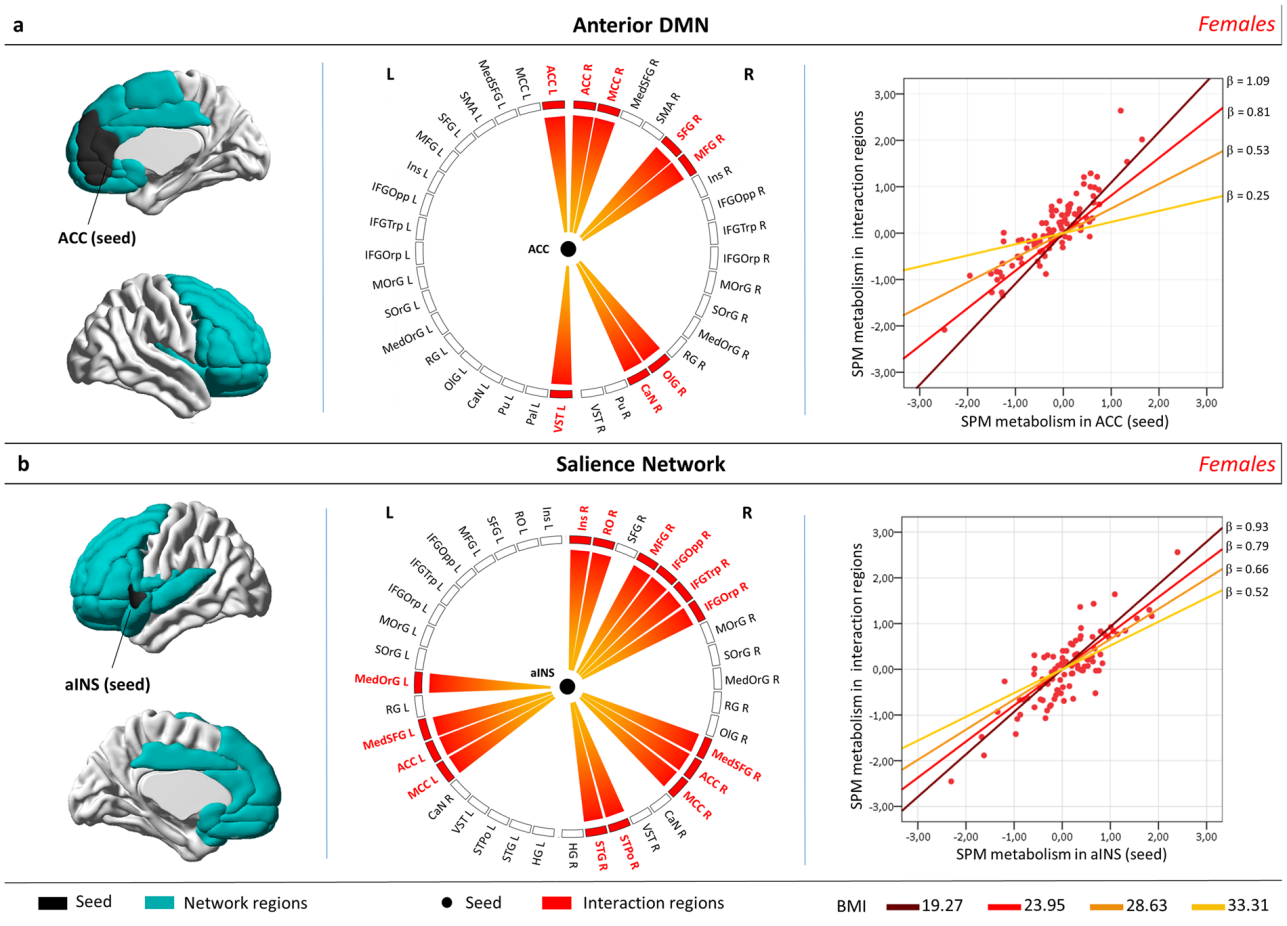
Significant modulatory effects by BMI level on brain metabolic connectivity of aDMN (**a**) and Salience Network (**b**), in female AD patients. Left panel: Panel showing, in blue, the brain regions belonging to the aDMN (**a**) and Salience Network (**b**). Networks’ seeds (i.e. ACC for aDMN and aINS for Salience Network) are depicted in black. The left side of the brain is shown on the left in the figure. Middle panel: Connectograms show brain regions belonging to each network. The central white dots denote networks’ seeds, i.e. ACC for aDMN and aINS for Salience Network. Networks’ regions whose connectivity is significantly modulated by BMI (i.e. interaction regions), are depicted in red (voxel-level p < 0.05 FDR-corrected, k = 100; cluster-level p < 0.05 FWE corrected). A significant modulatory effect by BMI level on metabolic connectivity is present for limbic and frontal regions related to reward processing and executive control. Right panel: the graph shows the BMI effects on metabolic connectivity. SPM contrast values in the network’s seeds (ACC for aDMN, aINS for Salience Network) on the x axis; SPM contrast values in the relevant fronto-limbic regions (interaction regions) on the y axis. The slope beta, representing the strength of metabolic connectivity between the network’s seed and the interaction regions, changes at different BMI levels. An increase of BMI corresponds to a reduction of the connectivity strength in both aDMN and Salience Network. Results are displayed on a 3D brain template using Brainnet toolbox^48^. Abbreviations: aINS, anterior insula; ACC, anterior cingulate cortex; CaN, caudate nucleus; Ins, insula; MCC, middle cingulate cortex; HG, Heschl’s gyrus; IFGOpp, inferior frontal gyrus, pars opercularis; IFGOrp, inferior frontal gyrus, pars orbitalis; IFGTrp, inferior frontal gyrus, pars triangularis; MedOrG, medial orbitofrontal gyrus; MedSFG, medial superior frontal gyrus; MFG, middle frontal gyrus; MOrG, middle orbitofrontal gyrus; OlG, olfactory gyrus; Pu, putamen; RG, gyrus rectus; RO, Rolandic operculum; SFG, superior frontal gyrus; SMA, supplementary motor area; SOrG, superior orbitofrontal gyrus; STG, superior temporal gyrus; STPo, superior temporal pole; VST, ventral striatum.

We tested whether there were significant modulation effects by BMI levels on connectivity strength in the main brain resting-state networks, i.e. the anterior and posterior Default Mode Networks (aDMN, pDMN), the Executive Control Network, the Attentional and the Salience Networks, the Limbic and Reward Networks. In the whole AD group, BMI had no effect on metabolic connectivity in any of the examined resting-state networks. When considering gender, however, females with higher BMI scores showed a lower connectivity between regions of the aDMN (specifically the anterior and right middle cingulum, right superior and middle frontal cortex, right olfactory cortex, left ventral striatum and right caudate nucleus; voxel-level p < 0.05 FDR-corrected, k = 100; cluster-level p < 0.05 FWE corrected; N = 94) and in the Salience network (specifically in the anterior and middle cingulum bilaterally, superior medial frontal gyrus, medial orbitofrontal gyrus, right insula, right rolandic operculum, right temporal pole, right superior temporal gyrus, right middle and inferior frontal gyrus; voxel-level p < 0.05 FDR-corrected, k = 100; cluster-level p < 0.05 FWE corrected; N = 94) (Fig. 3). No significant effect of BMI on brain networks’ connectivity was found in the male subgroup.

There were no significant interaction effects between BMI and either age, education or APOE status on brain metabolic connectivity in any resting-state network, in neither the whole AD group nor in the gender subgroups.

## Discussion

This is the first study exploring the effect of BMI on brain metabolism and connectivity, as major parameters of neural functionality, in a large sample of AD patients and also considering gender. Our findings reveal a negative effect of high BMI on brain metabolism in female AD patients. At comparable clinical severity, AD females with higher BMI levels showed less severe hypometabolism in AD-typical brain regions and reduced metabolic connectivity in fundamental resting-state functional networks. This suggests that, at comparable dementia severity, females with high BMI can withstand a lower degree of brain dysfunction and show greater vulnerability of brain networks, all the above reflecting reduced brain reserve mechanisms. Thus, we suggest that high BMI may exert a detrimental effect in the brain of female AD patients.

**Figure 4:**
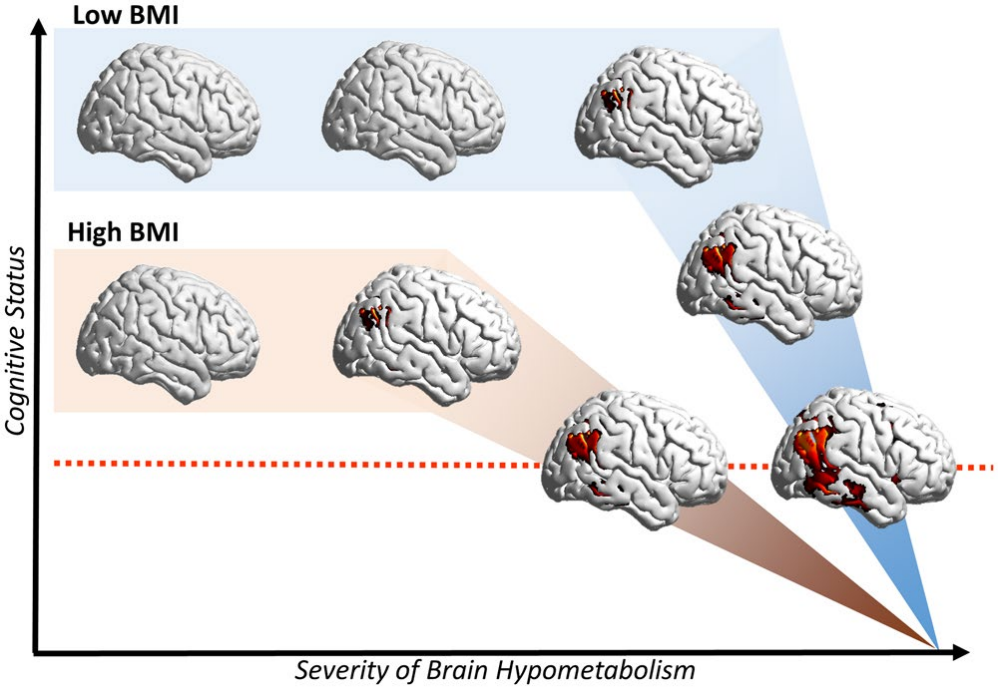
Pictorial representation of BMI effects on brain hypometabolism, hypothesizing a trajectory of cognitive decline and brain hypometabolism across females with low BMI and high BMI. Brain hypometabolism is represented on the x axis, cognitive status is depicted on the y axis. The dotted red line denotes cognitive status of AD patients with overt dementia, as in the present study. At this time point, severity of hypometabolism is significantly lower in high BMI as compared to low BMI AD females (p < 0.05 FDR-corrected, k = 100 voxels), in spite of comparable cognitive status, as measured by MMSE. This suggests that, in females with high BMI, a lesser amount of neurodegeneration is enough to cause dementia symptoms. Results are displayed on a 3D brain template using Brainnet toolbox^48^.

Our results suggest that a high BMI influences neurodegeneration and clinical manifestations in AD. Although AD neurodegeneration causes a reduction of brain metabolism in all patients, females with high BMI showed less brain hypometabolism than female with low BMI that was enough to cause comparable cognitive deficits and dementia (see Fig. 1). This result was also confirmed by the presence of a significant positive correlation between BMI and brain metabolism, controlling for age, disease duration, years of education and level of cognitive impairment. It may thus be conceived that, at a comparable degree of clinical severity, high BMI makes female AD patients to tolerate a lower load of neuropathological changes (Fig. 4). These findings are in agreement with the brain reserve theory^16^ originally described to explain the discrepancy between the quantity of neuropathology and the clinical expression in AD^18^. In several studies, life experiences have been reported as involved in the modulation of brain plasticity and structural changes in both healthy subjects and neurodegenerative conditions^16^. In spite of the absence of thorough information about occupation and other socioeconomic variables in our database, data on education were available, and these were controlled for. Of note, a previous study reported no significant effects of socioeconomic status on the association between education and risk for Alzheimer’s Disease^21^, also suggesting that education represents a much more important factor than socioeconomic status in Alzheimer’s disease.

Here, we show that a high BMI can be detrimental, causing less tolerance to underlying AD pathology, whereas a low BMI may contribute to neuroprotective mechanisms in dementia, particularly in females. Considering that the underlying AD pathology affects all the patients, it might be conceived that a high BMI leads to worse cognitive outcomes in the presence of AD neurodegeneration than low BMI. This interpretation is also supported by many studies which described a negative correlation between BMI levels and cognitive functioning during healthy lifetime^3,6,12^, and by the limited MRI structural evidence in healthy aging^22–25^, MCI and AD patients^26,27^ reporting an association of high BMI with reduced volumes of several brain regions, such as frontal, temporal, and occipital lobes, and brainstem.

The second main result of the present research study concerns the effects of BMI on brain metabolic connectivity, which is based on the assumption that regions whose metabolism is correlated are functionally interconnected^28^. Notably, only in the female AD group, high BMI, in comparison to low BMI, was associated with reduced metabolic connectivity of aDMN and Salience Network, in limbic and frontal regions (i.e. insula, anterior cingulum, medial frontal regions, orbitofrontal cortex and ventral striatum), crucially involved in reward processing and executive control (Fig. 3). In agreement with this result, two previous functional-MRI studies, exploring the functional brain connectivity in a group of obese adults, found decreased connectivity in the right anterior cingulate cortex and left insula^29,30^. These regions are crucially implicated in the rewarding effects of food, and the disruption of the reward-saliency circuit is indeed thought to be a key mechanism in obesity, increasing the reinforcing value of food and weakening control circuits^29^. Thus, we suggest that the above reported dysfunction in brain regions, which are related to reward mechanisms and executive control in obesity^31^, also reflects the detrimental effect of obesity on brain functioning in dementia. Of note, in a previous study, we found these specific brain regions to be crucial for brain reserve in both female AD patients and female healthy elderlies^18^. Specifically, the two female groups showed significant correlations between levels of brain reserve and brain metabolism in anterior-limbic and executive brain regions^18^. These results suggest that, in females, these anterior brain structures are mainly involved in brain compensatory processes to cope with both aging and dementia^18^. Considering all the above evidence, we suggest that high BMI could exert a detrimental effect on brain reserve also contributing to the vulnerability of neural systems selectively involved in protective mechanisms in both healthy aged and AD females^18^.

The significant effect of BMI on both brain metabolism and metabolic connectivity was not found in male patients. Despite similar obesity rates between sexes^32^, differences exist in the adverse effects of obesity on the brain, which seem to be exaggerated in women (see for review^33^). Although only few studies have explored gender differences in the relation between obesity and dementia risk, they suggest with some coherence that negative effects of obesity on cognitive functioning and AD risk are stronger in women than in men^33^. In particular, obese women show an increased disruption of blood-brain barrier^34^, temporal lobe atrophy^35^ and myelin degeneration^36^, resulting also in an increased rate of AD risk.

While there is some evidence for a gender effect on the obesity-related AD risk, the nature of this relationship is not completely understood. ApoeE4 and inflammation seem to be particularly involved, as they are associated with AD risk and obesity, showing stronger negative effects in females than in males (see for review^33^). Other factors influencing sex differences in obesity and dementia, such as insulin resistance, need to be considered. There is recent evidence for an association between the metabolic marker insulin resistance and AD (see for review^37^). The disturbances in glucose metabolism and insulin resistance were shown to be associated with neurodegeneration, accelerated cognitive decline and increased dementia risk^37^. Since insulin resistance is directly correlated with BMI, it may be the case that females with higher BMI are the most insulin-resistant, with important detrimental effects on the brain reserve in dementia. Unfortunately, the retrospective design of the present study did not allow us to include such a biomarker in the analysis.

Our study provides a first neurobiological evidence of the effect of high BMI levels on brain dysfunction in AD dementia, with female/male differences. Other biological and social factors in interaction with BMI may lead to gender-specific reorganization of brain functional networks, also limiting the neuroprotective mechanisms in dementia, which are in need to be further explored.

## Methods

### Subjects

We included 206 subjects with a clinical diagnosis of probable AD according to standard diagnostic criteria^38^ (females/males: 94/112; age: 72.27 ± 7.78 years; years of education 12.95 ± 4.38; disease duration: 3.72 ± 2.38 years; MMSE: 21.24 ± 4.03). They were recruited from two databases: 88 from the San Raffaele Hospital (Milan, Italy) and 118 from the Alzheimer’s Disease Neuroimaging Initiative (ADNI) database (http://adni.loni.usc.edu/). The ADNI was launched in 2003 as a public-private partnership, led by Principal Investigator Michael W. Weiner, MD. The primary goal of ADNI has been to test whether serial magnetic resonance imaging (MRI), PET, other biological markers, and clinical and neuropsychological assessment can be combined to measure the progression of mild cognitive impairment and early AD.

The AD diagnosis was also supported by the presence of the AD-like brain hypometabolism pattern, as assessed in single subjects with an optimized and validated ^18^F-FDG-PET SPM procedure^39^. All patients met diagnosis for probable AD as defined by National Institute on Aging-Alzheimer’s Association and described in McKhann *et al*.^38^. This implies absence of stroke and major psychiatric disorders (i.e. depression), as stated in the ADNI exclusionary criteria. Patients included in this study did not suffer from diabetes, with the exception of six male subjects. We also check the information about glycaemia at the time of PET scan, which was less than 160 mg/dl in all patients, including those six with diabetes (as recommended by the international guidelines)^40^.

Demographic characteristics are reported in Table 1 for the whole AD group and gender subgroups that were compared by means of two-sample independent t-tests. BMI levels were significantly higher in the male group as compared to the female group (t = 3.742, p < 0.001), at consistently with the higher prevalence of obesity and overweight reported in males by epidemiological studies in US and Europe (e.g. *Behavioral Risk Factor Surveillance study* (US); *Health Survey for England* (UK); *Osservasalute* (Italy)). However, BMI followed a normal distribution in both females and males groups, with kurtosis and skewness far below 1.96 in each group (females: Kurtosis = 1.150(SE = 0.493); Skewness = 0.859(SE = 0.249)); males: Kurtosis = 0.668(SE = 0.453); Skewness = 0.774(SE = 0.228)).

**Table 1:** Demographic characteristics (mean ± standard deviation) for whole AD group and gender subgroups, and significance of t-test for gender comparisons.

	Whole Group (N = 206)	Females (N = 94)	Males (N = 112)	P Value
Age (years)	72.27 ± 7.78	70.96 ± 7.93	73.36 ± 7.51	0.03^a^
Disease Duration (years)	3.72 ± 2.38	3.69 ± 2.45	3.74 ± 2.32	0.87
MMSE	21.24 ± 4.03	20.77 ± 4.51	21.64 ± 3.56	0.12
Education(years)	12.95 ± 4.38	12.16 ± 4.02	13.61 ± 4.57	0.02^a^
BMI (kg/m2)	24.99 ± 4.51	23.69 ± 4.44	26.08 ± 4.30	0.001^a^

Abbreviations: MMSE, mini-mental state examination; BMI, body mass index.

^a^Significant difference at the two-sample t-test comparing males and females.

Subjects provided written informed consent, following detailed explanation of each experimental procedure. The study was performed in compliance with the Declaration of Helsinki and it was approved by San Raffaele Hospital Ethical Committee.

### ^18^F-FDG-PET data acquisition and preprocessing

For the Nuclear Medicine Unit database of San Raffaele Hospital, the PET scan acquisition was performed according to the European Association of Nuclear Medicine guidelines^40^, while for the ADNI database the acquisition procedure is described in the “ADNI PET technical procedures manual, version 9.5” (http://adni.loni.usc.edu/wp-content/uploads/2010/09/PET-Tech_Procedures_Manual_v9.5.pdf). We created a single averaged 15-minute frame for each image starting at 30 or, in the majority of cases, at 45 minutes after radio-ligand injection. In this way, the uptake time for ADNI images was consistent with that of San Raffaele images. Intensity normalization was achieved by dividing each image by its global mean, in order to reduce inter-subject and inter-scanner variability^41^. Single-subject images were processed according to an optimized and validated SPM procedure^39,42^, to obtain individual hypometabolism SPM-t-map by means of a two-sample t-test, with the patient’s image being tested against a large database of 112 normal controls, (p < 0.05 with Family Wise Error (FWE) correction for multiple comparisons; minimum cluster extent set at k = 100 voxels). The resulting single-subject hypometabolism pattern was used as support for the diagnosis.

### Statistical analyses

#### Group analyses

Patterns of relative hypometabolism were then tested in each gender subgroup, split according to BMI (cut-off = 25; i.e. Low BMI (BMI <25); High BMI (BMI >25)). Demographics characteristics for each group are reported in Table 2. Significant differences in relative hypometabolism across BMI subgroups were assessed separately for females and males, by means of a two-sample t-test. Results were considered significant at p < 0.05 FDR-corrected at the voxel level. Only clusters with a minimum extent of 100 voxels and remaining significant at p < 0.05 FWE-corrected at cluster level were considered.

**Table 2:** Demographic characteristics (mean ± standard deviation) for gender and BMI subgroups.

	Females with low BMI (N = 61)	Females with high BMI (N = 33)	Males with low BMI (N = 53)	Males with high BMI (N = 59)	P Value
Age (years)	70.38 ± 8.42	72.12 ± 7.05	73.03 ± 8.56	74.01 ± 6.89	0.112
Disease Duration (years)	3.74 ± 2.68	4.01 ± 2.29	3.65 ± 2.28	4.04 ± 2.46	0.827
MMSE	19.94 ± 4.49	22.05 ± 3.86	20.81 ± 3.81	22.12 ± 2.45	0.001^a^
Education(years)	12.5 ± 4.15	12.7 ± 3.75	13.82 ± 4.55	14.21 ± 4.58	0.155
BMI (kg/m2)	21.28 ± 2.40	28.63 ± 3.48	22.68 ± 1.83	29.18 ± 3.36	<0.001^b^

Abbreviations: MMSE, mini-mental state examination; BMI, body mass index.

Significant difference at post-hoc comparison (MANOVA test) between:

^a^females with low BMI vs. males with high BMI.

^b^females with low BMI vs. males/females with high BMI; females with high BMI vs. males/females with low BMI; males with low BMI vs. males/females with high BMI.

We also explored the interaction effects of BMI and gender, along with age, education and APOE status on brain metabolism by means of multivariable regression models. Significance level was set at α = 0.05, with two-tailed significance tests.

#### Metabolic correlation analyses

We performed separate correlation analyses for the whole AD group and the gender subgroups. As ROI, we used the mask obtained from the group-level ^18^F-FDG-PET hypometabolism SPM map, thresholded at a highly conservative statistical significance (p < 0.05 FWE-corrected for multiple comparisons; k = 100 voxels), as described above. Next, the mean ^18^F-FDG uptake value from the ROI was computed for each patient and linearly correlated with BMI scores. Age, disease duration, MMSE and years of education were then included as nuisance variables in order to factor out their effects.

#### Metabolic connectivity analysis

The effect of BMI on the brain metabolic connectivity of the resting-state networks was investigated in the whole AD group and likewise separately for females and males. In particular, we applied the voxel-wise interregional correlation analysis in SPM, as described by Lee *et al*.^43^. The main assumption of this method is that brain regions whose glucose metabolism is correlated at rest are functionally associated^28^. The seed regions for each considered resting-state network were defined according to Shirer *et al*.^44^ (http://findlab.stanford.edu/functional_ROIs.html), Seeley *et al*.^45^ and Tomasi *et al*.^46^. In particular, we considered as seeds the anterior cingulate cortex for the aDMN^44^; the posterior cingulum and precuneus for the posterior DMN^44^; the left and right middle frontal and superior frontal gyri for the Executive Control Network^45^; the left and right parietal cortex for the Attentional Network^46^ and the anterior insula bilaterally for the Salience Network^45^; the amygdala for the Limbic Network^46^. Finally, the Ventral Striatum for the Reward Network was delineated following basal ganglia boundaries as provided by the AAL and manually-drawn on a high-resolution T1 anatomical template in accordance to the guidelines by Tziortzi *et al*.^47^.

The mean ROI counts were entered as variables of interest in a General Linear Model in SPM5 testing for voxel-wise whole-brain correlations in the whole AD group and also separately for females and males. Age and gender were considered as nuisance variables. For each resting-state network, we considered the pattern obtained by these correlation analyses as an explicit mask to test the effect of BMI on the metabolic connectivity of each resting-state network, for the whole AD group and again separately for females and males. Results were considered significant at p < 0.05 FDR-corrected at the voxel level. Only clusters with a minimum extent of 100 voxels and remaining significant at p < 0.05 FWE-corrected at cluster level were considered.

Lastly, we also explored the interaction effects of BMI with age, education and APOE status on the brain metabolic connectivity in each resting-state network by means of multivariable regression models.
